# Targeting Artemis Sensitizes B Cells to Topoisomerase 2 Poisons by Disrupting DNA-PKcs-Dependent Repair

**DOI:** 10.21203/rs.3.rs-6317145/v1

**Published:** 2025-06-19

**Authors:** Melissa L. Folkerts, Cameron Hom, Angie Nguyen, Kaiyuan V. Shen, Valeria Rangel, Pedro Ortega, Rémi Buisson, Selma Masri, Noritaka Adachi, Katheryn Meek, Nicholas R. Pannunzio

**Affiliations:** 1 Divison of Hematology/Oncology, Department of Medicine, University of California, Irvine, Irvine, CA, USA; 2 Department of Biological Chemistry, University of California, Irvine, Irvine, CA, USA; 3 Institute for Immunology, University of California, Irvine, Irvine, CA, USA; 4 Chao Family Comprehensive Cancer Center, University of California, Irvine, Irvine, CA, USA; 5 Graduate School of Nanobioscience, Yokohama City University, Yokohama, Japan; 6 Department of Microbiology, Genetics, and Immunology, Michigan State University, East Lansing, MI, USA

**Keywords:** NHEJ, DNA repair, Artemis, topoisomerase poison, DNA-PKcs, ATM, B cell, peposertib (M3814)

## Abstract

Topoisomerase 2 (Top2) poisons are widely used in cancer therapy but are associated with toxicity and secondary malignancies. Removing Top2 adducts requires endonuclease activity and repair by non-homologous end joining (NHEJ). We show that the NHEJ enzyme Artemis is a promising target for co-treatment with Top2 poisons. Inhibition of the Artemis activator, DNA-PKcs, with peposertib (M3814) sensitizes B cells to Top2 poisons while ATM or ATR inhibition does not. Interestingly, while M3814 treatment blocks Artemis endonuclease activity, Artemis phosphorylation is still detectible and is only affected upon inhibiting ATM, suggestive of an additional role for Artemis in DNA damage response signaling. Additionally, Artemis loss results in a significant accumulation of Top2 DNA adducts following treatment, indicating Artemis may act outside its canonical role in NHEJ to reduce adduct burden. Clinical data demonstrates that high Artemis expression correlates with poor survival in several cancers, and we demonstrate that Artemis function is critical for survival following combination drug treatment. These insights can be leveraged to unlock new avenues for the treatment of aggressive cancers by enhancing the cytotoxicity of agents through blockade of DNA break repair.

## INTRODUCTION

Combination therapies that both increase the load of DNA double-strand breaks (DSBs) and impair DSB repair hold promise for cancer treatment yet still pose a danger to untransformed cells^1,2^. Topoisomerase 2 (Top2) poisons act by covalently trapping the Top2 enzyme to DNA and generating DNA DSBs as replication and transcription machinery attempt to bypass the lesion. Top2 poisons such as etoposide and the intercalating anthracyclines doxorubicin and mitoxantrone have been a mainstay of cancer treatment for decades^3,4^. While the latter two have strong clinical relevance, the associated cardiotoxicity limits the dose that can be used^5,6^. Furthermore, treatment has been associated with the development of specific secondary leukemias unrelated to the primary cancer^4^. Combining Top2 poisons with compounds that impair DNA DSB repair may increase the efficacy of these drugs at lower doses and minimize toxic side-effects.

Several potential DNA repair targets, such as the DNA damage response phosphatidylinositol 3-related kinases (PIKKs) Ataxia Telangiectasia Mutated (ATM), Ataxia Telangiectasia and Rad3-Related (ATR), and DNA-dependent Protein Kinase Catalytic Subunit (DNA-PKcs), have overlapping roles in signaling for both homologous recombination (HR) and non-homologous end joining (NHEJ)^7^. As NHEJ is the dominant repair pathway in mammalian cells^8^, targeted therapies that reduce its efficiency would be ideal. As such, inhibitors of DNA-PKcs have been a major focus in the clinic with the drug peposertib (M3814) showing high specificity and being featured in several clinical trials^2,9,10^.

Unlike previous generations of kinase inhibitors, M3814 has been shown to be highly specific for DNA-PKcs^11^. Recent structural work has demonstrated that M3814 effectively blocks the kinase domain of DNA-PKcs by penetrating deep into the ATP binding pocket^12^. This prevents subsequent autophosphorylation at the ABCDE domain and further phosphorylation events at the PQR domain to hold DNA-PKcs in a basal state^13,14^. One presumed consequence of this is prevention of downstream activation of other enzymes that rely on DNA-PKcs kinase activity, such as the Artemis endonuclease^15^. Artemis plays a critical role in V(D)J recombination and has also been demonstrated to be a general NHEJ factor that is important for processing incompatible DNA DSB ends to prepare them for ligation^16–18^. Such ends often arise following exposure to radiation or chemotherapeutics. This suggests that inhibition of Artemis might enhance chemotherapy, however, there are currently no available inhibitors that specifically target Artemis at levels that would be clinically relevant^19^.

Artemis deficiency has been shown to contribute to tumorigenesis in several cancers, particularly if combined with a p53 deficiency^20,21^. Following transformation, high levels of Artemis expression have been attributed to poorer outcomes and increased resistance to treatment^19^. Thus, in normal cells, Artemis may act as a tumor suppressor that is important for maintaining genome stability through both its role in DNA repair and, possibly, through DNA damage response (DDR) signaling^22,23^. In contrast, abundant Artemis may allow for enhanced repair in cancer cells treated with radiation and chemotherapies leading to treatment resistance and secondary cancers.

It has been proposed that treatment of B cell acute lymphoblastic leukemia (ALL) may most benefit from development of a specific Artemis inhibitor as these cells are most dependent on Artemis to open covalently closed hairpin DNA DSB ends created by the recombination activating gene (RAG) complex during V(D)J recombination^19^. ALL cells have high expression of RAG, meaning the cells experience a high frequency of DSBs that uniquely require Artemis for processing. Specific targeting of this pre-B cell population would spare mature B cells and reduce the effects of therapies that indiscriminately target all white blood cells. Additionally, it is possible that other cancers that present with high Artemis levels would benefit from combination therapies that reduce Artemis activity while also inducing DNA DSBs. This is particularly relevant considering the number of blood cancers with recurrent translocations involving the immunoglobulin heavy (*IGH*) chain locus that occur at the pre/pro B cell stage as this is a focal point of Artemis activity^24–26^.

As a highly specific and potent Artemis inhibitor is still lacking^19,27^, we wished to explore if using M3814 to target upstream DNA-PKcs activity would serve a similar function in B cells also treated with Top2 poisons. Surprisingly, we found that M3814 treatment effectively phenocopies an Artemis-null genotype, even though Artemis phosphorylation is still detected. Conversely, an inhibitor of the ATM kinase measurably reduces Artemis phosphorylation but has no effect on viability following Top2 poisoning and minimal effects on hairpin opening. Furthermore, loss of Artemis leads to increased accumulation of Top2 covalent cleavage complexes on DNA. These results show that Artemis has a much larger role in maintaining genome integrity than previously thought. It can not only directly act on DNA to remove barriers to NHEJ repair but also acts in DDR signaling due to interactions with DNA-PKcs and ATM^28^. Inhibition of hairpin opening activity correlates with sensitivity to Top2 poisons and an Artemis truncation that lacks the C-terminal tail, the major target for Artemis phosphorylation^29^ is unable to complement this loss. This demonstrates the complexity of Artemis, its importance as a biomarker for aggressive cancer, and the potential for future targeted therapy.

## RESULTS

### Artemis plays a major role in repair of Top2-mediated damage

**Figure 1.**
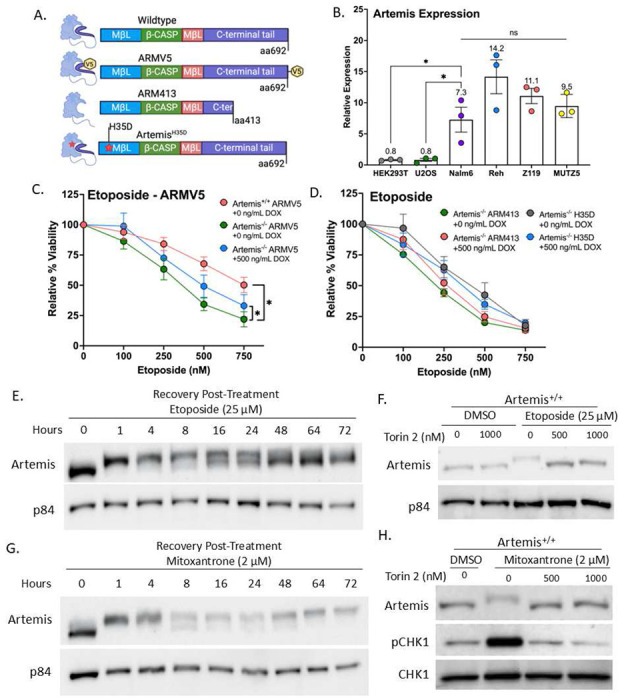
Artemis is vital for cell survival after treatment with Top2 poisons. (A) Schematic of cell lines virally transduced with a Dox-inducible cassette utilized in this study. Artemis protein is depicted to the left of the respective representative amino acid structure. (B) Bar plots showing relative Artemis mRNA levels in indicated cell lines (unpaired t-test; *p-value ≤ 0.05, ns=not significant). (C) Line graph comparing viability measured using CellTiter Glo in Nalm6 transduced with ARMV5 after 48h treatment with indicated concentrations of etoposide. Values are normalized to DMSO (paired t-test; *p-value < 0.05). (D) As in (C) but transduced with ARM413 or Artemis^H35D^ in Artemis^−/−^ Nalm6. (E) Western blot showing phosphorylated Artemis after etoposide treatment in Artemis^+/+^ Nalm6. Time points represent when lysates were collected after 1h etoposide treatment, washing with PBS, and recovery in drug-free media. p84 is used as a loading control. (F) Western blot showing inhibition of phosphorylated Artemis after 3h incubation with Torin 2 and 1h incubation with etoposide in Artemis^+/+^ Nalm6. p84 is used as a loading control. (G) As in (E), but with mitoxantrone. (H) As in (F), but with mitoxantrone and antibodies for phosphorylated CHK1 and basal CHK1.

To understand the role of Artemis in response to Top2 mediated damage, we introduced Doxycycline (Dox) inducible Artemis variants into both Artemis^+/+^ and Artemis^−/−^ Nalm6 pre-B cell lines^30^ via lentiviral transduction (Figure 1A). A major reason for performing these experiments in pre-B cell lines is to test the efficacy of Top2 poison combination therapy directly in a human ALL system. Furthermore, Artemis expression in pre-B ALL cell lines Nalm6, Reh, Z119, and MUTZ5 is significantly higher compared to other commonly used cell lines like the osteosarcoma cell line U2OS and embryonic kidney cell line HEK293T (Figure 1B). ARM413 has a truncation at amino acid position 413, resulting in the removal of the Artemis activation requirement following phosphorylation by DNA-PKcs at the C-terminal domain resulting in constitutive ARM413 endonucleolytic function^31,32^. ARMV5 is a full-length wild-type Artemis that has a C-terminal V5 tag. Histidine 35 of Artemis is important for coordinating the Zn^2+^ cofactor in the active site^13,27^. Artemis^H35D^ is a known human pathological variant that causes severe combined immunodeficiency (SCID) due to lack of Artemis function in V(D)J recombination^33^. Expression of these variants was verified by examining protein levels via western blot using lysates from transduced cell lines after overnight treatment with Dox (**Figure S1**).

To assess the effect of Artemis in response to Top2 poisons, we measured the viability of cells exposed to etoposide using CellTiter Glo. Building on prior observations^30^, after 48 hours of treatment with increasing concentrations of etoposide, Artemis^−/−^ cells were significantly more sensitive to the treatment than the Artemis^+/+^ cells (Figure 1C). For consistency, this was performed in Nalm6 cells with the Dox-inducible ARMV5 and we verify that ARMV5 is not detectable by western blot without Dox treatment (**Figure S1A**). With addition of 500 ng/mL of Dox, we measured a significant partial rescue of etoposide sensitivity (Figure 1C). In addition to CellTiter Glo, these results were also verified by flow cytometry by monitoring AnnexinV and intercalation of propidium iodide (PI) in Nalm6 cells lacking the ARMV5 cassette (**Figure S2A**). We also confirmed that etoposide sensitivity is a feature of mulitple human ALL cell lines by treating Reh, Z119, and MUTZ5 cells with increasing concentrations of the drug. MUTZ5 was isolated from a patient with the aggressive Philadelphia-like (Ph-like) ALL subtype^25,34^, and we were particularly interested in its drug sensitivity. Similar to Nalm6, the three additional cell lines demonstrated approximately 50% viability between 250 to 500 nM of etoposide with Reh showing slightly more sensitivity (**Figure S2B**).

Expression of the truncated ARM413 failed to rescue the etoposide sensitivity in Artemis^−/−^ cells as the viability was not significantly different with or without Dox treatment (Figure 1D). Truncations in this range have been shown to confer constitutive endonuclease activity to Artemis, though perhaps at a reduced rate^35^. In *Saccharomyces cerevisiae*, which lacks DNA-PKcs, ARM413 increases DSBs measured in a direct repeat recombination assay^32^ **(Figure S2C-S2D**), demonstrating that it is biologically active. This led us to speculate if the reduced viability following etoposide treatment was due specifically to Artemis endonuclease activity or if loss of the C-terminal tail was affecting the structure of the repair complex. To test this, we expressed the endonuclease dead Artemis^H35D^ variant in Artemis^−/−^ cells. We found that there was no difference with or without Artemis^H35D^ expression (Figure 1D), strongly suggesting that the lack of endonuclease activity is the major cause for decreased viability after etoposide treatment.

To rule out an etoposide-specific effect, we next determined sensitivity of Artemis^−/−^ cells with or without ARM413 expression to additional DNA damaging agents. Artemis-null Nalm6 cells are sensitive to ionizing radiation (IR) as we see a direct correlation between IR does and viability (**Figure S2E**) in contrast to previous reports that showed that loss of Artemis is less detrimental at higher IR doses^30^. Also, Artemis^−/−^ Nalm6 cells showed greater sensitivity to increasing concentrations of the Poly(ADP-ribose) (PARP) inhibitor olaparib than to either etoposide or IR (**Figure S2F**). Similar to the case with etoposide, ARM413 expression failed to rescue the IR and olaparib sensitivity (**Figure S2D and S2E**).

### Treatment with Top2 poisons results in Artemis phosphorylation

Given the enhanced sensitivity of B cells to Top2 poisons with an Artemis knockout, we also wanted to confirm that treatment resulted in phosphorylation and activation of Artemis. While multiple Artemis residues remain constitutively phosphorylated when grown in culture, several serines become phosphorylated following DNA damage leading to a molecule weight shift after protein modification^37^. Cells were treated with 25 μM etoposide for 1 hour, washed with PBS, cultured in drug free media, then collected at increasing time intervals with lysates analyzed for phosphorylated Artemis by western blot. At 1 and 4 hours after post-treatment recovery, we detect a clear shift to the higher molecular weight Artemis species (Figure 1E). While we see an accumulation of unphosphorylated Artemis starting at 8 hours, we still observe the presence of phosphorylated Artemis even at 72 hours after recovery. To further verify this change in Artemis was due to phosphorylation, we treated cells with Torin 2, which targets multiple PI3-kinase-related (PIKK) family members including DNA-PKcs, ATM, ATR, and mTOR ^38^. Torin 2 was added to cells 3 hours prior to 25 μM etoposide treatment and lysates were collected one hour after treatment. Torin 2 completely eliminated the molecular weight shift of Artemis following etoposide treatment (Figure 1F).

These results were consistent with those in response to another Top2 poison mitoxantrone (Figure 1G), overall demonstrating that treatment with Top2 poisons engages Artemis in the repair process. Interestingly with mitoxantrone, which is known to be a more potent Top2 poison, the higher molecular weight form remained the dominant form up to 72 hours. We also verified that Torin 2 treatment ablated this molecular weight shift as well as reduced the levels of phosphorylated Chk1 (Figure 1H), emphasizing its role as a potent kinase inhibitor. From these results, we see that Artemis both plays a role in cell survival after damage and responds to Top2 mediated damage, therefore providing evidence towards the efficacy of targeting Artemis for combination therapies.

### M3814 enhances sensitivity to Top2 poison but does not block Artemis phosphorylation

**Figure 2.**
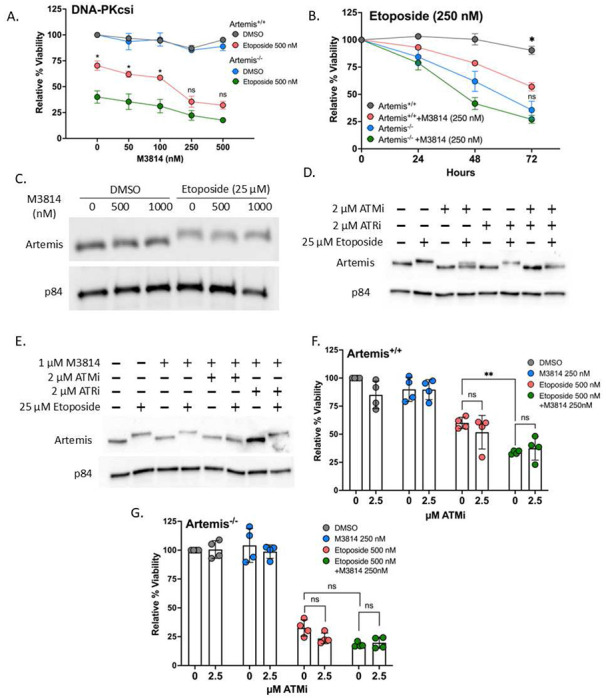
Inhibition of DNA-PKcs enhances toxicity of Top2 poisons. (A) Line graph comparing viability measured using CellTiter Glo. Values are normalized to DMSO (2way ANOVA, *=p value ≤ 0.05, ns=not significant). (B) Time course showing decreased viability over indicated time points after etoposide treatment with or without M3814 (2way ANOVA; *p-value = 0.0261(relative to Artemis^+/+^ +M3814)). (C) Western blot depicting Artemis phosphorylation after 3-hour treatment with M3814 and 1-hour treatment with etoposide. p84 is used as a loading control. (D) Western blot depicting Artemis phosphorylation after 3h treatment with ATMi (AZ32), ATRi (AZD6738), or both and 1h treatment with etoposide. p84 is used as a loading control. (E) As in (D), but with DNA-PKcsi (M3814). (F) Bar plots showing normalized viability to DMSO of Nalm6 Artemis^+/+^ measured using CellTiter Glo. Cells were treated with ATM inhibitor for 3h before treatment with etoposide for 24h (2way ANOVA; ** p-value = 0.0058). (G) As in (F), but with Artemis^−/−^ Nalm6.

Since we observed that Artemis plays a vital role in the response to Top2 mediated damage, we wanted to determine the potential of utilizing Artemis as a target for combination therapies with Top2 poisons. Thus, we assessed the effect of inhibiting DNA-PKcs through treatment of M3814 on cell viability in response to the Top2 poison etoposide. Given the many roles DNA-PKcs has in the DDR, this has the potential to impact several pathways, including the downstream activation of Artemis^39^. Using CellTiter Glo to measure cell viability after 500 nM etoposide treatment in response to increasing concentrations of M3814, we observed that treatment of etoposide sensitizes Artemis^+/+^ cells to the point that at 250 nM of M3814 they are not significantly different from Artemis^−/−^ cells (Figure 2A). This both demonstrates the potential of using M3814 in combination treatments to increase cell death to what is measured in Artemis null cells and that an Artemis-specific inhibitor without the pleiotropic effects of M3814 could also potentiate cytotoxicity.

To further assess the potential of using M3814 in combination therapies with Top2 poisons to increase effectiveness of chemotherapy, we studied whether the addition of M3814 could confer a reduction in viability with a lower etoposide dose. We exposed Artemis^+/+^ and Artemis^−/−^ to 250 nM etoposide with and without the addition of 250 nM M3814, then measured the viability in 24-hour intervals until 72 hours post-treatment. In Artemis^−/−^ cells, there was no significant difference in viability with combined treatment of M3814 and etoposide (Figure 2B). Strikingly, co-treatment of Artemis^+/+^ cells with 250 nM of etoposide and 250 nM of M3814 reduced cell viability to similar levels measured in Artemis^−/−^ cells within 72 hours. Expression of constitutively active ARM413 had no impact on cell viability, confirming its inability to rescue cytotoxicity when DNA-PKcs is compromised (**Figure S3A**). Treatment with 250 nM of M3814 alone without addition of etoposide had no effect on viability (**Figure S3B**) indicating its cytotoxicity presents following DNA damage. The three additional ALL cell lines Reh, Z119, and MUTZ5 also showed a significant decrease in viability with co-treatment of 500 nM etoposide and starting at the 250 nM M3814 concentration (**Figure S3C-S3E**). M3814 only began to affect viability at the highest 1 μM dose tested. This suggests that the addition of M3814 can enhance cancer cell killing effects in combination with Top2 poisons, potentiating the effectiveness of this combination in treating ALL.

Next, we wanted to address whether M3814 effectively blocks phosphorylation of Artemis in response to etoposide resulting in the significant decrease in viability. We treated Artemis^+/+^ cells with increasing concentrations of M3814 up to 1000 nM for 3 hours before a 1-hour treatment with 25 μM etoposide and visualized the effects via western blot. Interestingly, we observed that treatment with M3814 does not appear to prevent Artemis phosphorylation as there is no change in the molecular weight shift following etoposide treatment (Figure 2C). Thus, while DNA-PKcs inhibition with M3814 disrupts Artemis functionality, perhaps via misalignment at DNA DSB ends, these results suggest that other PIKKs can still phosphorylate Artemis and that DNA-PKcs autophosphorylation itself may be a more critical factor in downstream Artemis activity.

### ATM inhibition affects Artemis phosphorylation but not viability when combined with etoposide

The observation that M3814 did not effectively block phosphorylation of Artemis in vitro led us to assess other PIKKs previously described to activate Artemis to determine their role in response to DNA damage, including ATM and ATR^28,40,41^. Using previously described inhibitors, AZ32 (ATMi)^42^ and AZD6738 (ATRi)^43^, we found that 2 μM of ATMi treatment before a one-hour treatment with etoposide consistently and clearly reduced Artemis phosphorylation (Figure 2D). Thus, similar to IR^44^, there is an Artemis phosphorylation event that is dependent on ATM following exposure to Top2 poisons. This was not the case for the ATRi as there was a complete shift to the higher molecular weight product following etoposide treatment (Figure 2D). Combined treatment with both ATMi and ATRi greatly reduced the phosphorylation shift. Consistent with our findings above, M3814 alone did not affect phosphorylation (Figure 2E), however, combining M3814 with ATMi further removed the residual high molecular weight band present with ATMi treatment alone if we compare Lane 4 in Figure 2D and Lane 6 in Figure 2E, which is consistent with observations suggesting that both kinases have non-redundant repair functions at DNA DSBs^45^. This effect was comparable to the effect seen with Torin 2 or a combination of all three PIKK inhibitors (**Figure S3F**).

Since ATM has the ability to phosphorylate Artemis in response to etoposide, we wanted to test if ATM inhibition would also affect viability similar to M3814 (Figure 2A). Surprisingly, there was no significant change in viability when 2.5 μM ATMi was combined with 500 nM etoposide treatment in Artemis^+/+^ cells (Figure 2F). Only M3814 treatment appears to affect viability as we again observe a significant decrease in cells treated with both 500 nM of etoposide and 250 nM of M3814. The combination of 2.5 μM ATMi plus 250 nM M3814 also failed to further decrease viability from that measured with M3814 alone with etoposide treatment (Figure 2F). Similarly, in Artemis^−/−^ cells, neither treatment with M3814, ATMi, nor M3814 plus ATMi can significantly change viability measured with Artemis loss alone (Figure 2G). These results strongly suggest a separation of function between Artemis as a signaling molecule phosphorylated by multiple kinases and its direct role in affecting DSB repair and viability following treatment with DNA damaging agents. While DNA-PKcs kinase ability needs to be active to maintain viability in an Artemis-dependent manner, the role of Artemis phosphorylation by ATM is unclear.

### Co-treatment with ATM and DNA-PKcs inhibitors decrease hairpin opening

**Figure 3.**
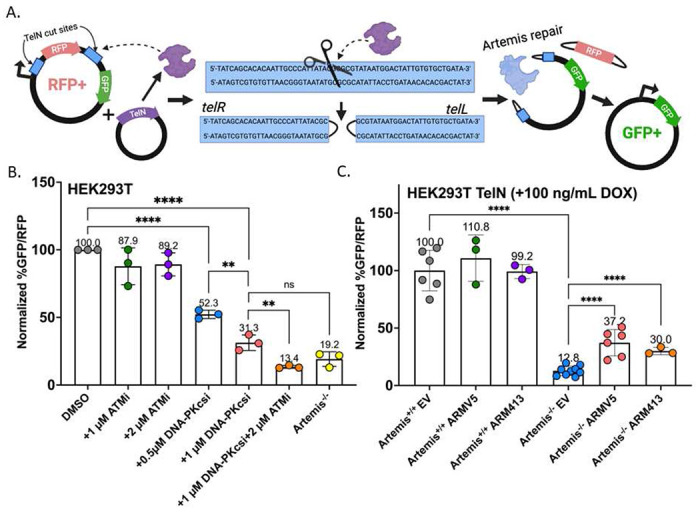
Inhibition of DNA-PKcs and ATM reduces Artemis-dependent hairpin opening. (A) Visual representation of hairpin assay as described in Meek, 2020^47^. Image created with biorender.com (B) Bar plot showing %GFP/RFP positive HEK293T cells after 72h transfection with plasmids shown in (A) normalized to Artemis^+/+^ with DMSO and treated with inhibitors at the same time as transfection (unpaired t-test; **** = p-value <0.0001, ** = p-value ≤ 0.01, ns=not significant). (C) Bar plot showing %GFP/RFP positive HEK293T cells 72h after transfection with Artemis variant plasmids and plasmids depicted in (A) normalized to Artemis^+/+^ (unpaired t-test; **** = p-value < 0.0001).

The most significant function of Artemis in NHEJ is opening hairpin structures at DNA ends, as the loss of Artemis endonuclease function results in a SCID phenotype due to the inability to repair RAG-induced DSBs^11,33^. While Artemis processing is not limited to hairpins^46^, using an assay where this function is specifically measured allows us to determine the extent to which kinase inhibition affects its activity. To address this, we have utilized a hairpin opening assay where the TelN Protelomerase is used to generate covalently closed DNA hairpin ends following cleavage at a specific 56 bp recognition sequence^47^ to test the effect of the PIKK inhibitors. This assay utilizes a TelN expression plasmid and an episomal substrate with TelN recognition sequences flanking an E2-Crimson (RFP) coding sequence (Figure 3A). TelN expression (**Figure S4A**) and cleavage excises the RFP sequence and leaves a linearized plasmid with hairpinned DNA ends. Following opening of the hairpins by Artemis, the ends can be repaired by NHEJ. This places the former RFP promoter upstream of a ZsGreen (GFP) sequence. Thus, the readout of hairpin opening efficiency is the percent of GFP positive cells divided by the percent of RFP positive cells (**Figure S4B**).

Due to the requirement for a cell line with high transfection efficiency, we chose to perform the assay in Artemis^+/+^ and Artemis^−/−^ HEK293T cells^47^. It has been shown that an Artemis knockout in HEK293T reduces recombination activity on hairpin DSB ends, validating the specificity of this hairpin assay to Artemis activity^47^. Since this assay is reliant on the availability of Artemis and its endonuclease activity, PIKK inhibitors that prevent kinase activity required for Artemis endonuclease function should also greatly reduce recombination^48^. What we observed with concentrations of M3814 as low as 0.5 μM and 1 μM was a significant 50% and 70% reduction in recombination activity, respectively, compared to the DMSO control (Figure 3B). Strikingly, even though ATMi reduced Artemis phosphorylation, neither a 1 μM nor a 2 μM treatment significantly affected hairpin opening (Figure 3B). Interestingly, co-treatment with ATMi and M3814 did significantly reduce recombination from 31.3% with M3814 alone to 13.4% with both drugs used together, which is comparable to levels in Artemis^−/−^ cells (Figure 3B). Since this is not measured with ATMi alone, inhibition of DNA-PKcs clearly has the most significant impact on hairpin opening whereas any role for ATM in this process is likely minimal.

Next, we wanted to address if the Artemis truncation, ARM413, can contribute to hairpin opening activity. By western blot, we determined that Dox was not required to initiate low levels of expression in Artemis^−/−^ HEK293T cells, leading to protein at similar endogenous levels measured in Artemis^+/+^ cells. Addition of 100 or 500 ng/mL of Dox greatly increased protein levels **(Figure S4C-S4D**). We found that, consistent with previous results, a knockout of Artemis in HEK293T led to a significant reduction in recombination activity (Figure 3C). When the ARMV5 variant was transfected into Artemis^−/−^ HEK293T, we saw a significant increase in recombination. ARM413 expression was able to partially rescue recombination in Artemis^−/−^ cells comparable to what is seen with ARMV5 (Figure 3C). This is consistent with previous findings in DNA-PKcs deficient CHO-V-3 cells where neither ARM413 nor full-length Artemis rescue V(D)J recombination activity and rescue only is measured upon co-transfection with a DNA-PKcs expression vector^31^. While the ARM413 truncation eliminates most of the regulatory region including residues that interact with the catalytic N-terminus^49^, major contact points between DNA-PKcs and Artemis at residues K368 to Y407 are still present^13^, meaning that interaction between ARM413 and DNA-PKcs is still possible.

### Artemis loss results in an accumulation of Top2-DNA adducts

**Figure 4.**
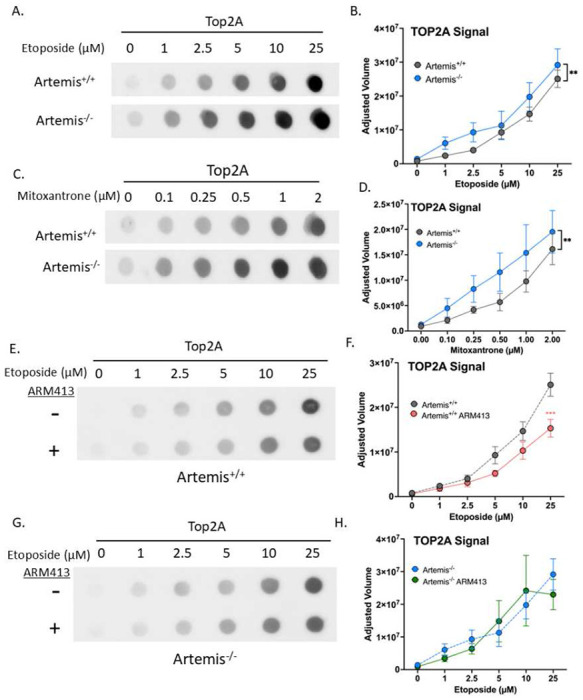
Artemis endonuclease activity removes covalently bound Top2A. (A) Dot blot showing Top2A signal in Nalm6 cells treated with etoposide at increasing concentrations for 1h. (B) Line graph of adjusted volumes from ImageLab software analysis (paired t-test; ** p-value = 0.0055). (C) As in (A), but with increasing concentrations of mitoxantrone. (D) Line graph of adjusted volumes from ImageLab software analysis (paired t-test; ** p-value = 0.008). (E) As in (A), but with Artemis^+/+^ Nalm6 and indicated expression of ARM413 treated overnight with 500 ng/mL Dox before treatment with etoposide. (F) Representative line graph of Top2A signal comparing Artemis^+/+^ cells treated with or without 500 ng/mL Dox to express ARM413. Dashed lines indicate data presented in (B) for direct comparison (2way ANOVA; *** p-value = 0.0006). (G) As in (E), but with Artemis^−/−^ Nalm6 and indicated expression of ARM413 treated overnight with 500 ng/mL Dox before treatment with etoposide. (H) As in (F), but with Artemis^−/−^ cells.

The nature of Top2 poisons such as etoposide necessitates endonuclease activity to remove the remaining 5’-phosphotyrosyl covalent bonds left by trapped Top2 cleavage complexes^50,51^. These DNA DSB ends require further processing through NHEJ to create ligatable joints for DSB repair^52^. If Artemis were involved in the removal and repair of these Top2 adducts, we should see an accumulation of these adducts in the absence of Artemis activity. To visualize accumulated Top2 through antibody detection, we used the rapid approach to DNA adduct recovery (RADAR) and dot blot method^53^. Though mammalian cells express an A and B isoform of Top2, we focus on the Top2A isoform, as cancer cells that are highly proliferative would have increased dependence on Top2A^50,54^. We clearly demonstrate that a knockout of Artemis in Nalm6 leads to an increase in Top2A adducts one hour after treatment with etoposide (Figure 4A). Image analysis software was used to quantify the adjusted volumes based on the intensity of the chemiluminescent signal detected on the nitrocellulose membrane compared to background. This allowed us to determine that Top2A accumulation was consistent and significantly higher in Artemis^−/−^ Nalm6 cells versus in Artemis^+/+^ cells (Figure 4B). Furthermore, this phenotype is consistent with treatment of another Top2 poison, mitoxantrone (Figure 4C–D). This demonstrates the necessity of Artemis in the repair of Top2-mediated DSBs on the molecular level, congruent with its role in downstream effects such as cell survivability.

Importantly, these dot blot experiments were performed one hour after acute doses (1-25 μM) of etoposide, whereas the viability experiments were done at nanomolar levels of etoposide over 24-72 hours. While the latter administers a non-lethal dose of drug to allow for processing of Top2 adducts and recruitment of DNA-PK and other NHEJ components, the former allows measurement of Top2 adducts soon after exposure and likely prior to many adducts being converted to frank DSBs. Since the RADAR assay relies on α-Top2A recognition, accumulation occurs prior to proteasomal degradation of the Top2 adduct^55^. Thus, this increase in Top2 adducts in Artemis^−/−^ cells may represent a role for Artemis in processing the covalent lesions outside of its canonical role in NHEJ, especially as we know that Artemis is phosphorylated by ATM within this timeframe (Figure 2E). This provided us with another opportunity to test the activity of the ARM413 truncation in a context where loading, activation, and alignment by DNA-PKcs would not necessarily be required.

We used Dox to express ARM413 in Artemis^+/+^ and Artemis^−/−^ Nalm6 cells treated with increasing doses of etoposide and performed the dot blot analysis (Figure 4E). Strikingly, we found that when ARM413 was expressed in the Artemis^+/+^ cells, there was a significant decrease in Top2A signal, suggesting that the ARM413 endonuclease activity contributed to removal of adducts (Figure 4F). This significance only emerged at the higher concentrations of etoposide, suggesting that ARM413 can assist in removing Top2 adducts when they have accumulated to a high abundance in the cell. Intriguingly, we were unable to detect a similar decreased level of Top2A in the Artemis^−/−^ cells (Figure 4G–4H). This may either be due to the instability of ARM413 in Artemis^−/−^ cells (Figure S1) or that the absence of Artemis in these cells changes overall DDR signaling to favor other repair pathways^56^. Overall, we can conclude that Artemis endonuclease activity specifically contributes to the repair of Top2 adducts induced by Top2 poisons, though the moderate change suggests this is not the major role of Artemis.

### High Artemis expression correlates to poor cancer prognosis

**Figure 5.**
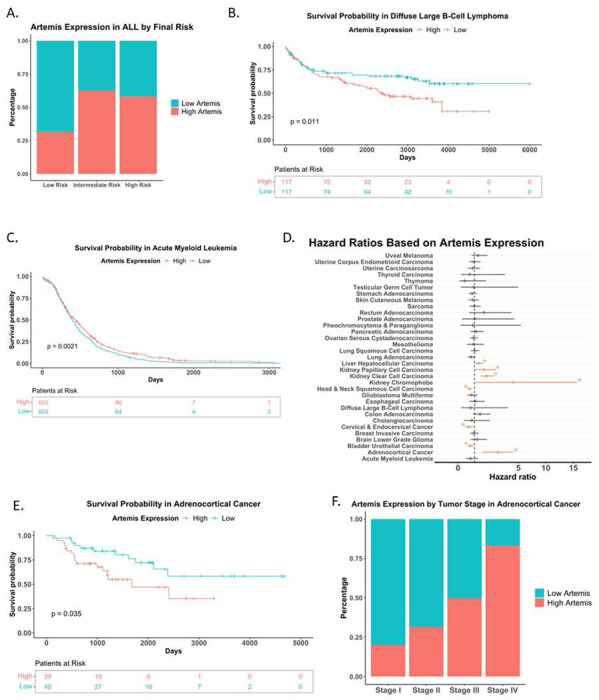
High Artemis expression correlates to poor prognosis in certain cancers. (A) Bar Chart comparing the percentage of patients within each DFCI Final Risk group with High/Low Artemis expression using the DFCI ALL database (n=12-24 patients per risk group). (B) Kaplan-Meier survival curve of Diffuse Large B-Cell Lymphoma Patients based on high versus low expression of Artemis using the Genomic Variation in Diffuse Large B Cell Lymphomas Dataset (n=234). (C) Kaplan-Meier survival curve of Acute Myeloid Leukemia Patients based on High/Low Artemis expression using the combined TARGET and TCGA AML cohorts (n=1,206). (D) Univariate cox proportional hazards model of the effect of Artemis expression on survival in the 33 cancer types in TCGA. (E) Kaplan-Meier survival curve of Adrenocortical Cancer Patients based on High versus Low expression of Artemis using TCGA (n=79). (F) Bar Chart comparing the percentage of patients with High/Low Artemis expression within each adrenocortical cancer tumor stage using TCGA (n=1-13 patients per risk group).

To provide insight into the clinical significance of Artemis inhibition, we assessed how expression levels impact cancer outcomes in patients, particularly those with blood cancers that involve recurrent translocations. It has previously been noted that high expression of Artemis reduces both overall survival and relapse free survival in pediatric ALL^19^. Using the Dana Farber Cancer Institute‘s (DFCI) cohort of ALL patients (16-001) under 22 years old, we were also able to conclude that high expression of Artemis correlates with high risk in ALL^57^ (Figure 5A). This is also the case when Artemis expression in ALL is evaluated against NCI Risk criteria^58^ where high risk is defined as white blood cell counts >50,000/mL and/or age >10 years old (Figure S5A). To determine if this was limited to B cell ALL, we also examined a diffuse large B cell lymphoma (DLBCL) patient cohort (n=234) from NCI^59^. Here too we found that high expression of Artemis significantly (p=0.011) reduced survival (Figure 5B). Interestingly, if we examine a cohort of acute myeloid leukemia (AML) patients taken from TCGA and TARGET (n=1206), we see the opposite trend where low Artemis levels result in significantly decreased survival (p=0.0021) (Figure 5C). This raises the possibility that high Artemis levels are more detrimental in lymphoid rather than myeloid malignancies.

Outside of a clear role in the pathology of lymphoid cancers, we also wanted to determine if other cancer types exhibited increased Artemis expression that correlated with survival. While determining the hazard ratios based on Artemis expression, we noted a clear dichotomy among cancers (Figure 5D). High Artemis expression significantly correlated with poor survival in adrenocortical cancer (Figure 5E), kidney clear cell carcinoma (Figure S5B), and hepatocellular carcinoma (Figure S5D). In contrast, like AML, some cancers actually had better survival rates if Artemis expression was high including cervical and endocervical cancer (Figure S5F), bladder urothelial carcinoma (Figure S5G), and head and neck squamous cell carcinoma (Figure S5H). Furthermore, we found that in several of these cancers high Artemis expression also correlated with advanced tumor stages. For example, approximately 20% of stage I adrenocortical cancer show high Artemis expression while this jumps dramatically to 80% in stage IV (Figure 5F). This was also true for kidney clear cell carcinoma (Figure S5C), but not for hepatocellular carcinoma (Figure S5E). Thus, in addition to Artemis corresponding to poor prognosis, in many cases there appears to be a role for Artemis in tumor progression as well. Overall, this clearly demonstrates that there are a broad range of cancers that may benefit from the application of targeting Artemis in therapeutic settings outside of blood cancers.

## DISCUSSION

The current state of ALL treatments emphasizes the need for new drug combinations to improve patient outcomes. While treatment of childhood B-cell ALL has allowed us to achieve disease-free survival (DFS) rates of over 90%^60^, we are still lagging in the treatment of adult ALL with DFS rates below 50%^61–63^. This is further compounded by the existence of aggressive subtypes of ALL, particularly Ph-like ALL^25,64,65^ where overall survival (OS) is below 25% with high rates of relapse. ALL patients are typically treated with one of two regimens, hyperfractionated cyclophosphamide, vincristine, doxorubicin, and dexamethasone (hyper-CVAD) or pediatric-inspired regimens (PIRs) that include a form of asparaginase^61,66^. People with genetic ancestries common in Latin America have been shown to have less favorable outcomes when treated with regimens containing asparaginase, which is significant as Ph-like ALL is more prevalent in this population^25,66^. Overall, neither treatment has greatly improved outcomes in adult ALL. Both hyper-CVAD and PIRs include some type of Top2 poison due to their effectiveness in treating blood cancers due to the high load of DNA DSBs that accumulate in these rapidly dividing cells. Thus, combining a Top2 poison with drugs that prevent DNA DSB repair would likely have high efficacy in treating ALL, but which factor and which DNA DSB repair pathway to target is not clear.

**Figure 6.**
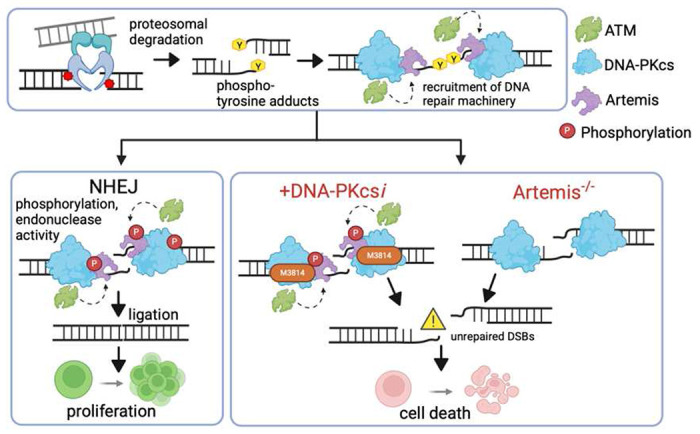
Artemis is required for efficient repair of Top2 poison-induced damage via NHEJ. Processing of covalently bound Top2 adducts leaves tyrosine residues bound to the 5’ phosphates of DNA that need to be removed by Artemis prior to ligation. In NHEJ Artemis is recruited to DNA DSB ends by DNA-PKcs and can be phosphorylated by DNA-PKcs as well at ATM, but physical interaction with DNA-PKcs is required for proper Artemis alignment for processing DNA ends. Treatment with the DNA-PKcs inhibitor M3814 does not prevent ATM-mediated phosphorylation of Artemis but blocks DNA-PKcs autophosphorylation that prevents conformational changes required to align Artemis with DNA ends for end processing resulting in unrepaired DSBs. Figure was created using biorender.com.

In this work, we clearly demonstrate that combination of the DNA-PKcs inhibitor M3814 combined with the Top2 poison etoposide leads to greater killing of B cells than etoposide alone. Experiments were also performed in Artemis^−/−^ to better understand the role Artemis plays in etoposide toxicity. Surprisingly, we found that the cytotoxicity in Artemis^+/+^ cells treated with etoposide and M3814 was not significantly different from etoposide treatment alone in Artemis^−/−^ cells. As repair of Top2 adducts has been shown to require NHEJ^14,56^, this demonstrates that Artemis is a critical factor in that repair and that one consequence of DNA-PKcs inhibition is blocking Artemis function (Figure 6). Future work on directly inhibiting Artemis activity may even further benefit cancer treatment regimens as it would not have the pleiotropic effects that come with blocking a major DDR kinase like DNA-PKcs. This could likely have benefits beyond co-treatment with Top2 poisons as Artemis^−/−^ cells are also sensitive to irradiation and PARP inhibition (Figure S2D-S2E).

In addition to a role for Artemis in repair of Top2 adducts via canonical NHEJ (Figure 6), our experiments revealed other possible functions of Artemis in B cells. Curiously, M3814 treatment did not eliminate the molecular weight change in Artemis consistent with phosphorylation while treatment with an ATM inhibitor completely eliminated it. Conversely, ATM inhibition had no significant effect on viability when combined with etoposide treatment, suggesting that the phosphorylation itself may be less critical than the interaction that Artemis has with DNA-PKcs. Further supporting this, we also found that a truncated form of Artemis missing the C-terminal tail, a major phosphorylation target, is unable to complement viability in Artemis^−/−^ cells treated with etoposide. Notably, a pathological Artemis variant that terminates the protein after residue 460 has been shown to decrease DNA repair efficiency and result in SCID, further emphasizing that the C-terminus is important for Artemis function^33,67^. Recent structural work demonstrates that interactions between the Artemis C-terminal tail and DNA-PKcs may be important for tethering the two proteins together while DNA-PKcs phosphorylation leads to conformation changes to algin Artemis for DNA cleavage^13^. Thus, even if the truncation retains endonuclease activity^32^, only full-length Artemis is able to function in NHEJ repair, at least when DNA-PKcs is present at DNA DSB ends. Previous work has demonstrated that in the absence of DNA-PKcs, neither full-length nor truncated Artemis endonuclease activity appears functional^31^.

We also verified that M3814 treatment can affect Artemis activity in a functional hairpin opening assay^47^. Artemis is essential for efficient opening of covalently closed hairpins at DSB ends^15,68,69^ and increasing concentrations of M3814 effectively eliminate this hairpin opening in Artemis^+/+^ cells. We note that ATM inhibition has minimal effects on hairpin opening. While cancer cells are continuously cycling, that the reduced phosphorylation of Artemis via ATM inhibition has little effect on viability and hairpin opening in these B cells points to NHEJ factors like DNA-PKcs and Artemis as being the more critical targets in blocking DNA repair in blood cancer treatments that rely on overloading cells with DSBs.

Further evidence of this is that we measure a clear and significant increase in the load of Top2 adducts covalently bound to DNA in Artemis^−/−^ cells. While one pathway of removing Top2 adducts is proteasomal degradation of the bulk of the Top2 protein followed by removal of the remaining tyrosine residue covalently bound to the 5’ end of DNA by Tyrosyl-DNA Phosphodiesterase 2 (Tdp2), a Tdp2-independent form of repair that involves the DNA DSB repair endonuclease complex consisting of Mre11/Rad50/Nbs1 (MRN) has also been described^14,55^. We propose that Artemis may be involved in an additional form of Tdp2-independent repair and that this may be cell-origin specific as pre-B cells express high levels of Artemis. Future studies will further define the role of Artemis in this pathway to better understand the impact on chemotherapeutic treatment of blood cancers. Overall, these results put Artemis at the nexus of the response to and the repair of DNA damage from Top2 poisons and can be leveraged to improve cancer treatments in not only B cell cancers, but cancers in other tissues^70,71^ where high Artemis expression correlates with reduced survival.

## RESOURCE AVAILABILITY

### Lead contact

Requests for information, resources, and data will be fulfilled by Dr. Nicholas R. Pannunzio (nrpann@hs.uci.edu)

### Materials availability

Cell lines and plasmids generated in this study are available upon request.

### Data and code availability

Clinical outcome correlations were generated using publicly available as referenced. No new code was developed in this study.

## MATERIALS AND METHODS

### Cell lines and cell culture

Nalm6, Reh, and Z119 were grown in RPMI-1640 supplemented with 1% Glutamax (Gibco), 1% penicillin/streptomycin, 10% FBS, and 50 μM β-mercaptoethanol. MUTZ5 cells were grown in the previously described conditions, but with 20% FBS. Nalm6 virally transduced with the Dox-inducible Artemis variants were under constant selection with 0.5 μg/mL G418. U2OS and HEK293T were grown in DMEM supplemented with 1% Glutamax (Gibco), 1% penicillin/streptomycin, and 10% FBS. HEK293T Artemis^−/−^ was grown in supplemented DMEM with 10 μM ciprofloxacin.

### Generation of stable cell lines expressing Artemis variants

Virus with each pINDUCER-20 Artemis variant plasmid was generated using HEK293T with a 3^rd^ generation lentiviral system. Nalm6 cells were transduced with the generated virus through spinoculation in complete media. Monoclonal cell lines were selected for with 2 mg/mL G418 and screened for expression of variants using western blot.

### Construction of Vectors

The cDNA of each variant described was integrated into pINDUCER-20 using gateway cloning and the Invitrogen Clonase II system per manufacturer’s instructions. Successful clones were confirmed with enzyme digestion and sanger sequencing through Azenta. Each vector was transformed into electrocompetent DH5α and expanded for MIDI-preps (Takara Bio).

### Cell Viability Assays

Cells were plated in 96 well clear-bottom plates and 500 ng/mL Doxycycline was added to sample wells indicated to express the respective construct. Plates were incubated overnight at cell culture conditions listed above. Cells treated with inhibitors were incubated for 3h with the described inhibitor before addition of Top2 poison. After respective incubation times (described in figure legends), cells were transferred to a white-wall, clear-bottom 96 well plate. CellTiter Glo was diluted 1:1 with PBS and added to each well. The plate was then placed in a VarioSkan plate reader and shaken for 2min with a subsequent 10min pause. Luminescence values were obtained and normalized to respective controls to represent viability changes with dose response.

### Cell Viability Time Course

Cells were seeded in 48 well plates and treated with 250 nM M3814 for 3h. 250 nM etoposide was then added and plates were incubated at above cell culture conditions. At 0, 24, 48, and 72h after etoposide treatment, 10 μL samples were taken from the same well for viability to be determined with Trypan Blue and the Countess 3 FL cell counting system. Viability percentages were recorded and normalized to respective 0h values.

### qPCR for Artemis Expression

Cells were grown under standard cell culture conditions before being lysed with Trizol. RNA was precipitated with isopropanol, pelleted, and washed with 70% ethanol. RNA was then quantified and 1 μg per sample was used for cDNA synthesis. cDNA was synthesized using Maxima H Minus cDNA synthesis master mix according to manufacturer’s instructions. cDNA was then diluted 10- and 500- fold for Artemis and 18s (reference for normalization) amplification, respectively. Samples were loaded in triplicate for technical replicates into MicroAmp Fast Optical 96-well Reaction plates with Power Up SYBR Green qPCR Master Mix and a comparative CT with melt curve qPCR was performed. Quality checks were conducted through Quant Studio (Thermo) before analysis. For calculation of expression, 18s technical replicate Cq values were averaged and subtracted from the respective Artemis Cq values. Final expression values for each technical replicate were then calculated with the following equation: Δ*Cq* = 2^−Cq^ × 1000. Technical replicates were averaged and plotted on described bar plots.

### Episomal Hairpin Assay

HEK293T cells or HEK293T Artemis^−/−^ cells were seeded at 25% confluency in a 24-well plate and incubated overnight at 37°C to allow cells to settle and adhere. For cells expressing the Artemis variants, 0.25 μg of variant-expressing plasmid was transfected with BioT, according to manufacturer’s instructions, and incubated for 8h with or without 500 ng/mL Dox in fresh DMEM with previously described cell culture conditions. Media was then replaced with fresh DMEM plus 500 ng/mL Dox before transfection of 0.25 μg of TelN-V5, 0.125 μg of TelN/Crim, or both using BioT. Cells were incubated overnight and media was replaced with DMEM plus 100 ng/mL Dox before flow cytometry acquisition 72h from the last transfection. To test the inhibitors, HE293T cells were transfected with 0.125 μg of TelN-V5, 0.5 μg of TelN/Crim, or both using BioT in DMEM. The PIKK inhibitors were added to the media at the same time as transfection, and media was replaced with DMEM plus each indicated inhibitor after overnight incubation. Flow cytometry acquisition was conducted 72h from transfection. For acquisition, 20,000 events were collected, viable cells were gated and then gated for single cells on the Novocyte Quanteon. Analysis was done with FlowJo by gating on histograms of PE-Cy5-H (for RFP positive) and FITC-H (for GFP positive). Successful recombination was quantified as %GFP/%RFP.

### RADAR and Dot Blot Assay

Cells were seeded with 500 ng/mL Dox added to sample wells indicated to express ARM413 and incubated overnight. Cells were then treated with indicated concentrations of etoposide or mitoxantrone for 1h before being spun down and lysed in DNAzol. DNA was precipitated with 100% ethanol and tubes were inverted multiple times until DNA was visible before incubating at −20°C overnight. DNA was then pelleted and washed with 70% ethanol. Pellets were then resuspended in 8 mM NaOH with gentle pipetting. DNA was left overnight at 4° C for full reconstitution.

DNA collected from the RADAR method was quantified using a NanoDrop (Thermo; ND-ONE-W). Dilutions of DNA to obtain 20 ng/μL in 200 μL were then calculated and made in water. Concentrations were confirmed on the NanoDrop again before adding 1x TBS to obtain a concentration of 10 ng/μL. 1 μg of DNA was diluted in 1x TBS for a total 200 μL loading volume. Nitrocellulose membrane (0.45 μm) and two thin filter papers were cut to the size of the BioDot Apparatus (BioRad) and equilibrated in 1x TBS for at least 20 minutes. TBS was pipetted into randomly selected wells to check for even flow. Unused wells were covered with tape and sample wells were washed twice with 200 μL TBS through vacuum flow. Samples were then run through the BioDot apparatus, and the membrane was washed with TBS for 2min before crosslinking at 1200 x100 μJ/cm^2^ and blocking with BLOTTO (5% non-fat milk in 1x TBST) for one hour. Membranes were then washed briefly with TBST and incubated overnight at 4°C with a 1:1000 dilution in BLOTTO of Top2A (Cell signaling; 12286). Membranes were then washed 3 times for 5min with 1x TBST and incubated at room temperature with a 1:10,000 dilution of Anti-rabbit (Millipore; 12-348) in BLOTTO.

### Western Blot

For suspension cells, cells were pelleted then washed with PBS before resuspending in PBS and an equal volume of 2x lysis buffer (117 mM Tris-HCl, 12% Glycerol, 3.6% SDS, 2mM DTT). For adherent cells, media was aspirated, and PBS was used to wash the wells. Cells were scraped with a 1:1 mix of PBS and 2x lysis buffer. Lysate samples were then boiled at 100°C for 5 minutes and spun down to remove debris. Protein concentration was quantified using a BCA protein assay kit (Thermo; 23227) and equal amounts of protein were loaded into each gel. A wet transfer method was used to transfer proteins to a nitrocellulose membrane. Membranes were blocked with BLOTTO for 1h before being probed with primary antibody. Antibodies used for the western blots were: Artemis (D708V) (1:1,000 in 5% BSA/TBST) (Cell Signaling, Cat# 13381) , V5-tag (D3H8Q) (1:1,000 in BLOTTO) (Cell Signaling, Cat# 13202), phospho-CHK1 (Ser345) (1:1000 in BLOTTO) (Santa Cruz Biotechnology, Cat# sc-8408), CHK1 (G-4) (1:1000 in BLOTTO) (Cell Signaling, Cat# 2348), (Mouse IgG (H+L) (1:3,000 in BLOTTO) (Thermo Fisher, Cat# 31430), and Rabbit IgG (1:10,000 in BLOTTO) (Thermo Fisher, Cat# 12-348).

### Direct Repeat Recombination Assay

Strains of *Saccharomyces cerevisiae*, as previously described^32^ contain a chromosomally integrated *URA3* gene flanked by 180 base pairs of homology for the *HIS3* gene allowing for selection after random DSBs and subsequent single-strand annealing (SSA). Strains containing either a plasmid coding for a galactose-inducible ARM413 or control plasmid were inoculated into galactose selection media lacking uracil and incubated for 5h with rotation at 30°C to log phase growth. After 72h, cells were plated onto selective media lacking histidine to select for positive CFUs and YPD for total CFUs. Recombination frequency was calculated as the number of positive CFUs divided by total CFUs.

### Flow Cytometry Viability Assay

Cells were seeded at 500,000 cells per mL with 500 ng/mL Dox added to sample wells indicated to express the respective construct and incubated overnight. Cells were then treated with etoposide at increasing doses for 24h. After treatment, cells were then spun down to pellet and resuspended in 100 μL Annexin V Binding Buffer (4 mM NaCl, 0.25 M HEPES, 1 mM CaCl_2_). Staining was then performed with a 1:1000 dilution of Annexin V-AF647 and Propidium Iodide (PI). For acquisition, 20,000 events were collected, viable cells were gated and then gated for single cells on the Novocyte Quanteon. Analysis was done with FlowJo by gating quadrants on APC (for Annexin V positive cells) and PE-Texas Red (for PI positive cells). Viability was recorded as the percentage of cells negative for both Annexin V and PI.

### ImageLab Quantification of Dot Blots

Each dot blot image was imported and analyzed in ImageLab software (BioRad, version 6.1.0 build 7). Briefly, equally sized circles were drawn around each dot using Volume Tools, including one to reference as “background.” The “adjusted volumes” representative of signal intensity with a localized background subtraction from separate biological replicates were recorded and used for subsequent analysis on GraphPad Prism software.

### Statistical Analysis

All statistical methods used are described in the figure legends. Statistics were calculated through GraphPad Prism software and conducted on 3 or more biological replicates. All bar plots and line graphs depict the mean with SEM error margins.

### Patient Data Analyses

Patient data from TCGA (The Cancer Genome Atlas), TARGET, and Genomic Variation in Diffuse Large B Cell Lymphomas Dataset (GDC NCICCR-DLBCL) were downloaded from UCSC Xena Data Browser. Normalized FPKM expression counts and sample metadata from Tran *et.al*, 2022 were downloaded from GEO (GSE 181157). Data was loaded into R (version 4.4.0) and stratified into two groups, High/Low Artemis based on the median expression value of Artemis (DCLRE1C) within each respective cancer type and dataset. Bar charts made via ggplot2 (version 3.5.1) were used to compare the number of High/Low Artemis patients across different tumor stages and risk classifications. Kaplan-Meier survival curves were generated using the survival (version 3.5-7) (https://cran.r-project.org/web/packages/survival/citation.html) and survminer (version 0.4.9) packages. Univariate Cox proportional hazard models were done on each of the 33 TCGA cancer types to determine which cancer types significantly associated Artemis expression with overall survival.
